# The landscape and predicted roles of structural variants in *Fusarium graminearum* genomes

**DOI:** 10.1093/g3journal/jkae065

**Published:** 2024-03-28

**Authors:** Upasana Dhakal, Hye-Seon Kim, Christopher Toomajian

**Affiliations:** Department of Plant Pathology, Kansas State University, Manhattan, KS 66506, USA; USDA, Agricultural Research Service, National Center for Agricultural Utilization Research, Mycotoxin Prevention and Applied Microbiology Research Unit, 1815 N University St., Peoria, IL 61604, USA; Department of Plant Pathology, Kansas State University, Manhattan, KS 66506, USA

**Keywords:** *Fusarium graminearum*, Oxford Nanopore sequencing, structural variation, structural rearrangements, recombination, transposable elements

## Abstract

Structural rearrangements, such as inversions, translocations, duplications, and large insertions and deletions, are large-scale genomic variants that can play an important role in shaping phenotypic variation and in genome adaptation and evolution. We used chromosomal-level assemblies from eight *Fusarium graminearum* isolates to study structural variants and their role in fungal evolution. We generated the assemblies of four of these genomes after Oxford Nanopore sequencing. A total of 87 inversions, 159 translocations, 245 duplications, 58,489 insertions, and 34,102 deletions were detected. Regions of high recombination rate are associated with structural rearrangements, and a significant proportion of inversions, translocations, and duplications overlap with the repeat content of the genome, suggesting recombination and repeat elements are major factors in the origin of structural rearrangements in *F. graminearum*. Large insertions and deletions introduce presence–absence polymorphisms for many genes, including secondary metabolite biosynthesis cluster genes and predicted effectors genes. Translocation events were found to be shuffling predicted effector-rich regions of the genomes and are likely contributing to the gain and loss of effectors facilitated by recombination. Breakpoints of some structural rearrangements fall within coding sequences and are likely altering the protein products. Structural rearrangements in *F. graminearum* thus have an important role to play in shaping pathogen–host interactions and broader evolution through genome reorganization, the introduction of presence–absence polymorphisms, and changing protein products and gene regulation.

## Introduction

Phenotypic trait variation arises due to small- (SNPs and small Indels) and large-scale (structural rearrangements) genomic variants, and these variants are key to genome evolution and speciation (Priest *et al*. 2020). Meiotic (and parasexual) recombination, DNA repair during meiosis and mitosis, transposon activities, and horizontal gene transfer are some of the mechanisms by which large-scale rearrangements originate in genomes (Mehrabi *et al*. 2017). Double-stranded DNA breaks generated during these processes are repaired using non-homologous end joining, which is an error prone method of DNA repair.

Studies on large-scale chromosomal rearrangements in fungal genomes have elucidated that structural rearrangements play an important role in fitness, adaptation, fungicide resistance, speciation, and evolution (Massonnet *et al*. 2018; Tralamazza *et al*. 2019; Wang *et al*. 2020; Langner *et al*. 2021). Chromosomal rearrangements have been implicated in presence–absence polymorphisms in secondary metabolite synthesis genes in *Phaeoacremonium minimum* (Massonnet *et al*. 2018) and the *Fusarium graminearum* species complex (FGSC; Tralamazza *et al*. 2019), as well as in the evolution of effector superfamily genes, host-specificity, and speciation in the genus *Taphria* (Wang *et al*. 2020). Similarly, in the wheat fungal pathogen *Zymoseptoria tritici*, a recent emergence of fungicide resistance is driven by chromosomal rearrangements (Badet *et al*. 2021). Likewise, in the powdery mildew pathogen of grape *Erysiphe nectar*, copy number variation in EnCYP51 genes is associated with demethylation inhibitor fungicide resistance (Jones *et al*. 2014). Dispensable mini-chromosomes, a special type of structural variation, most likely have emerged in *Magnaporthe oryzae* by the rearrangement and duplication of core chromosome segments and are implicated in adaptation and host-specificity (Langner *et al*. 2021).

Despite the important role of structural rearrangements in phenotypic trait variation, adaptation, and evolution, they have not been systematically studied in many fungi because high-quality contiguous assemblies for multiple isolates are lacking. The recent advent of long-read sequencing technologies has facilitated assembly of chromosomal-level assemblies at relatively low cost and should accelerate the study of structural rearrangements in fungal and other genomes. These high-quality genomes will also be useful resources for many other genomics analyses.

This study characterizes structural variation in the genomes of the plant pathogenic fungus *F. graminearum*. *F. graminearum* is a fungal pathogen of wheat and barley worldwide and causes Fusarium head blight (FHB) disease (McMullen *et al*. 1997; Goswami and Kistler 2004). Its genome is composed of four chromosomes and a mitochondrial genome (King *et al*. 2015), with distinct genomic regions evolving at different rates, fast and slow (Laurent *et al*. 2018; Wang and Zhang 2021). The “fast” genome is enriched in genes with roles in plant–microbe interaction, secondary metabolite synthesis, and genes expressed *in planta* during infection, and this region is key to adaptation (Laurent *et al*. 2018). It is part of the diverse FGSC, which is composed of several plant pathogens that can cause FHB and produce mycotoxins (Tralamazza *et al*. 2019; Munkvold *et al*. 2021). Multiple genome assemblies are available for FGSC species, yet besides *F. graminearum*, only two additional species, *Fusarium asiaticum* and *Fusarium meridionale*, have chromosome-level genome assemblies available at NCBI. Previous genome resequencing studies have defined the pangenome of *F. graminearum* and delineated core and accessory genes (Walkowiak *et al*. 2015, 2016; Kelly and Ward 2018; Machado *et al*. 2018; Alouane *et al*. 2021). Accessory genes from this and related FHB species are located in highly diverse, frequently recombining regions near the chromosomal ends and are differentially conserved among pathogen populations (Walkowiak *et al*. 2016; Kelly and Ward 2018). In the United States of America, *F. graminearum* exists in populations correlated to the dominant variant of trichothecene mycotoxin produced by the isolate, known as its chemotype. The populations NA1, NA2, SLA, and NA3 are imperfectly correlated to the major chemotypes 15ADON, 3ADON, NIV, and NX-2, respectively. The reference isolate PH-1 is a 15ADON isolate from the NA1 population, but chromosomal-level assemblies are lacking for the other chemotypes and populations. In this study, we assembled high-quality genomes of four representative isolates from different *F. graminearum* populations using long reads from Oxford Nanopore technology. We discuss the presence and distribution of these large-scale genomic variants as well as their potential role in survival, adaptation, and pathogen fitness.

## Materials and methods

### Origin of sequenced isolates

The four *F. graminearum* isolates sequenced and assembled here were collected from diseased wheat heads by other researchers and have been included in previous publications (Gale *et al*. 2007; Kuhnem *et al*. 2015). Supplementary Table 1 includes the date and location of collection for each isolate.

### DNA extraction, library preparation, and sequencing

Mycelia for DNA extraction were collected by growing the isolates on liquid complete media for 2 days and frozen at −80 °C. DNA was extracted using a published protocol (Schwessinger and McDonald 2017; Wang *et al.* 2021) with some modifications. Briefly, 500 mg of frozen mycelium was ground in liquid nitrogen with 5 g sand using a mortar and pestle. The powder was transferred to a 50 ml centrifuge tube with 14 ml of lysis buffer and 25 ul RNAse A (20 mg/ml) and mixed gently by inverting the tube. The tube was incubated on a rocking platform at slow speed for 1 h at room temperature. This was followed by the addition of 200 µl proteinase K and incubation for 1.25 h on the rocking platform at room temperature. The tubes were centrifuged at 5,000*×g* for 12 min at 4 °C. The supernatant was treated with phenol:chloroform:isoamyl mixture, incubated for 15 min (mixed gently by inverting during incubation) and centrifuged at 4000*×g* for 10 min at 4 °C. The aqueous layer was transferred to a new tube and phenol:chloroform:isoamyl treatment was repeated once. The aqueous layer was mixed with an equal volume of sodium acetate (NAOAc, 3M, pH 5.2) and DNA was allowed to precipitate at room temperature for 15 min. The tubes were centrifuged at 8000*×g* for 30 min at 4 °C to pellet the precipitated DNA. The pellet was washed twice with 70% ethanol and resuspended in 0.1M Tris-HCl (pH 8.5). The DNA was cleaned further by adding 12.5 µl RNAse A and incubating for 1 h on the rocking platform. This step was followed by phenol:chloroform:isoamyl treatment, precipitation of DNA with sodium acetate, and washing with 70% ethanol. In the final step, DNA was resuspended in 50 µl of 0.1M Tris-HCl at pH 8.5. High molecular weight DNA (DNA fragments longer than 30 kb) was selected using Blue Pippin followed by cleanup using AMPure XP (Beckman Coulter) beads before quantifying the DNA using Qubit. Loading solution and electrophoresis buffer provided with the Blue Pippin kit were used.

Each Oxford Nanopore sequencing library was prepared using ligation kit (SQK-LSK109) and about 1,000 ng genomic DNA. The SQK-LSK109 protocol was slightly modified. During DNA repair and end prep, incubation at 20 °C was increased to 30 min from 5 min. Similarly, during clean up with AMPure beads, the DNA sample was incubated for 10 min and the first wash was performed with 600 µl of 75% ethanol. Also, during adapter ligation, DNA samples were incubated for 30 min following mixing with adapters and ligation reagents and further incubated for 10 min after mixing the samples with AMPure beads. The libraries for individual isolates were loaded into separate MinION flow cells and sequenced.

### Base calling and genome assembly

Following sequencing, DNA bases were called using the Guppy base caller. Genomes were assembled using the Canu-1.9 assembler (Koren *et al*. 2017). minReadLength and minOverlapLength were set to 5,000 and 1,000, respectively. corOutCoverage was set to either 40 or 80. Default settings were used for the rest of the parameters. An updated Canu version (2.1.1) was used to reassemble the mitochondrial genomes. The completeness of each assembly was assessed using BUSCO v5.4.4 using the Hypocreales database (Simão *et al*. 2015).

### Error correction

Nanopolish _0.11.3 and Pilon version 1.24 were used to correct the draft assemblies (Walker *et al*. 2014; Loman *et al*. 2015). Nanopolish uses the signal of the raw fast5 reads to correct errors. Briefly, the raw fast5 sequences were indexed using nanopolish index and the draft genome assembly was indexed using minimap2 (Li 2018). The raw fast5 sequences were mapped to the draft genome assembly using minimap2. The resulting binary alignment map (BAM) file was converted to sequence alignment map (SAM) file format, indexed, and sorted. This was followed by splitting the assembly into 50 kb fragments. Each fragment was corrected by nanopolish and then all fragments were merged to produce the corrected assembly. Two rounds of nanopolishing were performed on each of the four genomes. The corrected assembly from the first round was used as the input for the second round of polishing. The first round of polishing produced about 92,000 changes while changes in the second round averaged about 900 per genome. Insertions accounted for over 90% of the changes made for all genomes.

The four isolates were also sequenced using the Illumina HiSeq platform to obtain short reads for Pilon. For DNA extraction, fungal mycelia was collected after growing the isolates in liquid complete media for 2 days and frozen at −80 °C. The mycelium was freeze-dried for 3 days. About 500 mg of the dried mycelia was ground to powder using a bead beater at 29–30 rpm for 3 min. DNA was extracted using a CTAB method. Briefly, 800 µl of warm 2% CTAB buffer and 10 µl of 2-mercaptaethanol were added to each sample and incubated at 65 °C for 30 min with occasional gentle mixing during the incubation. Chloroform:isoamyl alcohol mixture (24:1) was added and gently mixed by inverting the tubes. This was followed by centrifugation at 12,000 rpm for 20 min. The aqueous phase was transferred to a fresh tube and DNA was precipitated by adding cold (−20 °C) isopropanol. The DNA was pelleted by centrifuging the tube at 10,000 rpm for 5 min, air dried, and resuspended in 1× TE buffer. The RNA contamination was removed by treating DNA with RNAse A. DNA concentration was quantified using Qubit. An Illumina HiSeq library was prepared with Nextera adapters, and sequenced to obtain 150 bp paired reads. Illumina sequences were trimmed using ILLUMINACLIP in trimmomatic-0.39 (Bolger *et al*. 2014). Sequences shorter than 60 bp and leading and trailing bases below quality score 3 were removed. Reads were scanned in sliding windows of 4 bp and sliding windows with a mean quality score below 13 were dropped. The remaining paired-end reads were used for Pilon. Quality filtered Illumina reads were aligned to the nanopolish-corrected draft assembly using default parameters in BWA-MEM (Li 2013). The output BAM file was changed to a SAM file, indexed, and sorted. Pilon was used to correct errors with parameters –minmq 40, –fix bases, and –minqual 15.

In addition to making small insertions, deletions, and substitutions, Pilon flagged potential large assembly errors producing duplications that might require collapsing. Three rounds of Pilon polishing were performed on assemblies obtained after nanopolishing. The number of corrections decreased from round one to round three. Pilon made about 22,000 corrections per genome in the first round. Small insertional corrections were the largest followed by small deletions and substitutions. The second round of pilon polishing made about 100 changes while less than 10 corrections were made in the third round for all four assemblies.

### Genome annotation

The newly assembled genomes from four isolates, including nuclear and mitochondrial chromosomes as well as extra unplaced contigs not identified as contamination or duplicated, were annotated with Funannotate (Palmer and Stajich 2020). Publicly available RNA sequences SRR1179894, SRR1179897, and SRR9054433, collected from the wild-type PH-1 reference strain, were used to help with the gene prediction.

### Aligning orphan contigs from conspecific isolates to genome assemblies

The population genomics study of Kelly and Ward (2018) assembled sets of “orphan contigs” from sequencing reads of 60 isolates of *F. graminearum* that did not map to the PH-1 reference genome. The MUMmer tool NUCmer (nucmer –mum) was used to align the orphan contigs sets against each of the four genomes assembled in this study with default parameters, and the MUMmer tool show-coords was used to parse the alignment output (Marçais *et al*. 2018). The function count_overlaps from the Python package PyRanges (Stovner and Sætrom 2020) was used to generate alignment depth information across all 60 isolates along the four nuclear chromosomes from each of the four assemblies.

### Identification of structural variants

In addition to the four genomes we assembled, we downloaded chromosomal-level assemblies of three *F. graminearum* isolates, FG-12 (GCA_019343145.1), CML3066 (GCA_900073075.1), and CS3005 (NRRL 46420; GCA_000599445.1) from the NCBI Genome database. The software SyRI by Goel *et al*. (2019) was used to detect the structural variants (SVs) in seven *F. graminearum* genomes. Genomes were aligned to reference genome PH-1 (GCA_900044135.1) using the whole genome alignment tool MUMmer (Marçais *et al*. 2018). The MUMmer program NUCmer was used for the alignment with minimum cluster length (c), breaklength (b), and minimal length of maximum exact match (l) set at 100, 500, and 50, respectively. NUCmer identified all 50-mers, and 50-mers within 500 bp are merged to form a single alignment. Alignments were filtered to remove alignments that were less than 90% identical and shorter than 100 bp using the delta-filter tool in MUMmer. SyRI was then used to identify inversions, translocations, duplications, and indels, as well as SNPs and highly divergent regions (HDRs), using default settings. Only alignments to the four nuclear chromosomes of PH-1 were retained, and mitochondrial genomes were not compared. Chromosomes in the reference and query genomes were labeled to have identical IDs. SyRI first identifies the syntenic regions between the homologous chromosomes. Non-syntenic regions are reversed in one of the chromosomes to identify the inversions (reverse complementing one of the chromosomes makes the inverted regions syntenic). The remaining non-syntenic regions are then screened for translocations or duplications. A custom script was used to extract the genes (complete and partial) falling in the rearranged segments using the start and end position of the events in the reference PH-1 genome. The rearrangements that partially overlapped genes in PH-1 were manually checked to identify if the chromosomal break points overlapped coding sequence and if a gene falls within the rearranged segment.

Regions present in one or more query genome but absent in PH-1 (insertions relative to PH-1) were searched against a database of “orphan” protein sequences predicted by Kelly and Ward (2018) (their S2 file) to identify the potential genes present in the inserted segments. The database consists of genes present in one or more of the 60 isolates but absent in PH-1. The functions of the genes in the insertions closely matching the genes in the orphan protein database were identified by performing BLAST searches of the orphan proteins against the GenBank fungal protein database (Camacho *et al.* 2009). The presence of secretory signals in genes was assessed using the software SignalP 6.0 (Teufel *et al*. 2022).

We defined regions with high recombination based on the genetic map by Laurent *et al*. (2018). Coordinates of the regions were used to determine which structural rearrangements fall within the high recombination regions in *F. graminearum*. We also mapped the PH-1 reference genome to a reference genome of *Fusarium verticillioides* isolate 7600 (GCA_027571605.1) using the NUCmer program in MUMmer to identify ancient chromosomal fusion sites in the PH-1 genome. Overlap of ancient chromosomal fusion sites with regions of high recombination was observed.

Relatedness of the isolates was checked by constructing a neighbor joining tree with the nj function of the ape R package (Paradis and Schliep 2019) using single nucleotide polymorphisms in the query genomes identified by SyRI. To test for the enrichment of certain genomic features in certain genomic regions, or the enrichment of one feature in regions containing a different feature, we first partitioned the genome into 50 kb windows. Then, we recorded the presence or absence of transposable elements (TEs) and rearrangements (inversions, translocations, or duplications) in each window, and indicated whether each window was within 1 Mb of a chromosome end and/or corresponded to a high recombination region. We computed odds ratios from 2 × 2 contingency tables based on the genome regions/features across the 50 kb windows, and used Fisher's exact test to test the null hypothesis of an odds ratio equal to 1, i.e. independence.

### Detection of repeats and TEs

We used the extensive de novo TE annotator (EDTA) pipeline to characterize the repeat content and the presence of TEs in the genomes (Ou *et al*. 2019). EDTA combines the TE annotation programs LTRharvest (Ellinghaus *et al*. 2008), LTR_retriever (Ou and Jiang 2018), Generic Repeat Finder (Shi and Liang 2019), TIR-Learner (Su *et al*. 2019), HelitronScanner (Xiong *et al*. 2014), and RepeatModeler (Flynn *et al*. 2020) to identify the repeat elements. The elements are passed through basic and advanced filters of the program to produce a non-redundant TE library for annotation of structurally intact and fragmented TE elements. We supplied the pipeline with the coding sequences of the PH-1 reference genome which is used to purge gene sequences in the TE library.

## Results

### Genome assembly and annotation

We selected four *F. graminearum* isolates 23522, 23468, 23389, and 23473 for Oxford Nanopore sequencing (Supplementary Table 1). All are part of a larger population genomic study, and these four were chosen to span the *TRI* genotype and population diversity of that study. 23468 and 23473 are 15ADON isolates from North Dakota and Minnesota, respectively, but group distantly in a principal component analysis of a larger set of isolates (Dhakal *et al*. 2021). 23522 is a 3ADON isolate from Minnesota and 23389 is an NX-2 isolate from New York.

**Fig. 1.**
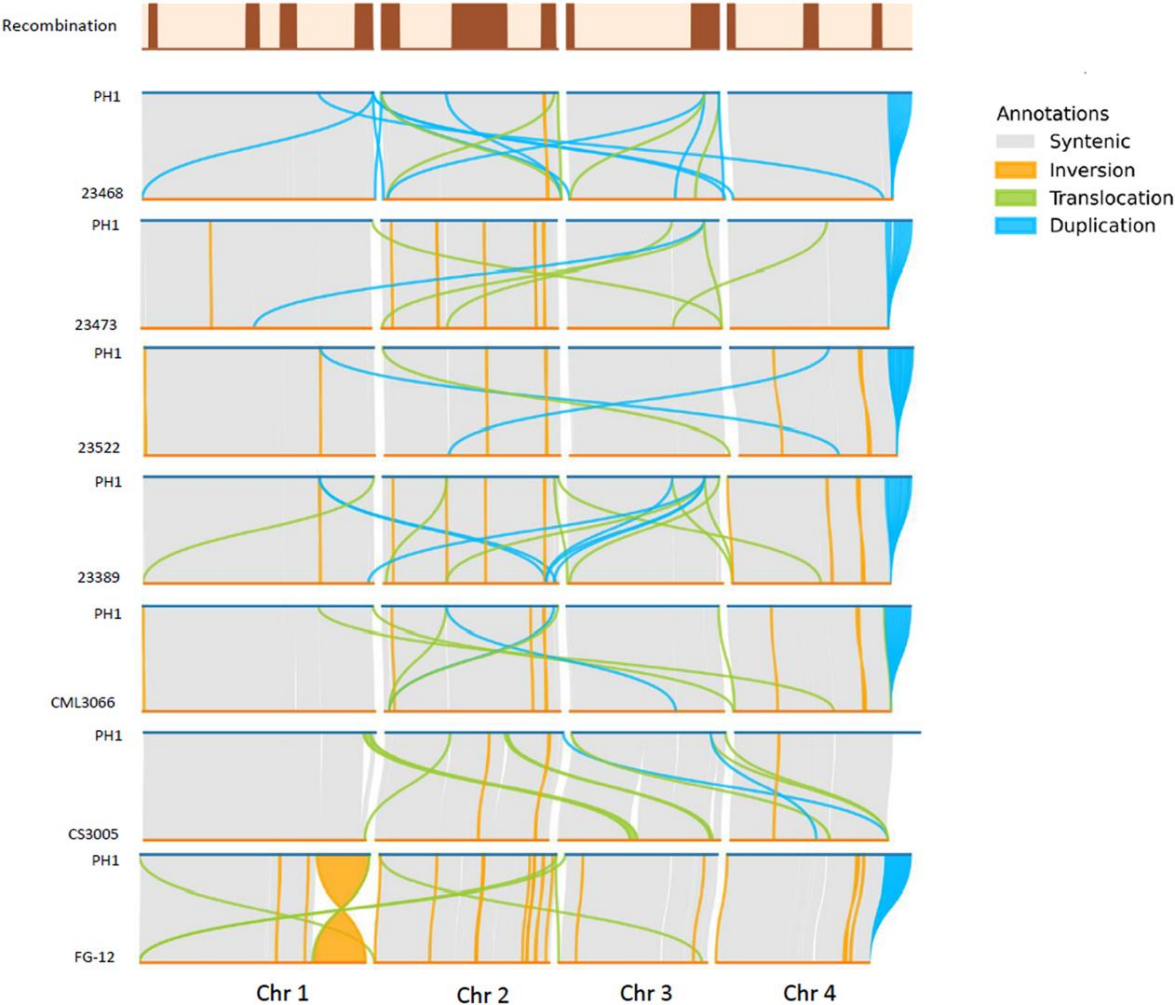
Chromosomal rearrangements in *F. graminearum* genomes. Rearrangements greater than or equal to 1,000 bp are plotted. The darker bars in the top plot represent regions with high recombination rates.

Oxford Nanopore sequencing produced a range of sequence data, from 4.68 to 10.14 Gb for the four *Fusarium* isolates (Table 1). This amount of raw sequence corresponds to 123× to 267× expected coverage, based on the 38.04 Mb size of the *F. graminearum* PH-1 reference genome. Assembling the genomes using Canu gave chromosomal-level assemblies for our four isolates. For each of the four assemblies, the four largest contigs represent nearly complete chromosomes, as evidenced by the detection of telomere repeat sequence at the majority (26 of 32) of contig ends and the finding that the total length of these four contigs always exceeded 96.5% of the total length of the four chromosomes from the PH-1 reference assembly (Supplementary Table 2). The region of ribosomal repeats in the subtelomeric region of chromosome 4 was not fully assembled into contigs in any of the Nanopore assemblies, accounting for four of the chromosome ends that are missing telomere repeats. The primary difference in chromosome length relative to PH-1 was due to the absence of most of the ribosomal repeat region in these assemblies (Fig. 1 and Supplementary Table 2).

**Table 1.** Summary statistics of *F. graminearum* genomes used in this study.

Isolate	Chemotype	Raw data (Gb)	Coverage	Contigs		Genome size	Nuclear genome size	BUSCO score
				Original	Post-cleanup			
23389	NX-2	6.07	160	33	17	43,661,591	37,046,483	96.2
23468	15ADON	7.3	192	8	8	37,441,186	37,004,440	97.7
23473	15ADON	10.14	267	10	5	37,080,489	36,786,251	97.7
23522	3ADON	4.68	123	6	5	37,374,555	37,126,805	97.7
CML3066	15ADON	NA	NA	5	NA	37,008,806	36,908,675	97.7
CS3005	15ADON	NA	NA	1205	NA	36,667,552	36,313,044	97.5
FG-12	15ADON	NA	NA	6	NA	35,897,492	35,851,270	NA

*Note*. Genome sizes are in base pairs. The top four assemblies were produced in this study.

We also obtained mitochondrial genomes for all four isolates. Mitochondrial genomes from the original assemblies were larger than expected. Reassemblies of the nanopore reads mapping to these contigs with a newer version of Canu identified for three of the isolates a large artificially duplicated segment to be trimmed. For the fourth isolate, a similar duplicated segment was identified using MUMmer when mapping the mitochondrial contig against itself. These duplicated sequences were manually removed from the polished mitochondrial contigs.

The genome assemblies produced by Canu consisted of 6–33 contigs (Table 1), each containing at least one contig that did not correspond to a full chromosome or mitochondrial genome. These extra contigs generally had poor read support and GC contents that did not match our chromosomal contigs. We removed one extra contig that was exceptional in its strong read support, a 4.7 Mb long circular contig from the assembly of isolate 23389, because it was 88% identical to the bacteria *Stenotrophomonas spp.* Additionally, the NCBI Foreign Contamination Screening Tool removed 20 contigs it identified as foreign contamination, and two additional contigs were removed in the process of performing gene annotation at the Funannotate Clean step because they had been marked as short segments duplicated from larger chromosome contigs. After this filtering, the 23389 assembly retained 12 extra contigs, while the 23468 assembly retained 3. The three extra contigs for 23468 are not only small (under 116 kb), but were assembled with only 11, 1, and 1 reads (Supplementary Table 2). The extra contigs from the 23389 assembly are similarly small (from 46 to 193 kb), and all but three were assembled with fewer than 35 reads. The GC content of these extra contigs also suggests which others could represent foreign contamination. The nuclear chromosome contigs had GC contents between 47% and 49%, and the mitochondrial contigs between 31% and 32%. The contigs removed from the 23389 assembly by the GenBank contamination tool all had GC contents >60%, while those removed from the 23473 assembly had GC contents between 43% and 44%. Two of the three extra contigs from 23468 are unlikely to represent nuclear chromosomal sequence as they have GC contents below 40%, while nine of the 12 extra contigs from 23389 are likely bacterial contaminants, with GC contents above 60% (Supplementary Table 2). The assemblies have an average and minimum BUSCO score of 97.3 and 96.2, respectively (Table 1). Funannotate identified 14,529, 13,670, 13,553, and 13,647 genes for 23389, 23468, 23473, and 23522, respectively.

### An expanded reference genome resource

The high variation in gene content in *F. graminearum* isolates from the same or different populations (Kelly and Ward 2018) highlights the weakness of using only a single reference genome in genomics analyses such as read mapping. In this previous study, reads from 60 isolates that did not map to the PH-1 genome were de novo assembled into orphan contigs, which added an average of 705 kb of extra sequence to each isolate's genome. The relative genomic location of these orphan contigs could not easily be determined using the PH-1 reference, but many contigs could be tentatively placed when compared with new reference genomes, especially ones from the same population. On average, over 486 kb from the orphan contigs could be mapped from each isolate to the “best match” assembly (the assembly that maximized mapping for an isolate; Supplementary Table 15). In particular, an average of 769 (and up to 962) kb from isolates assigned to STRUCTURE population NA2 mapped to the 23522 assembly, while an average of 519 (and up to 551) kb from isolates assigned to STRUCTURE population NA3 mapped to assembly 23473 (Supplementary Table 15). For isolates belonging to the same population as the PH-1 isolate (NA1), that reference genome performed relatively better, but even here on average an additional 267 (and up to 395) kb of contigs could be mapped to another assembly (Supplementary Table 15). The placement of the orphan contigs on the assemblies is also informative. The majority of them map to subtelomeric regions as predicted in the original study (Kelly and Ward 2018; Supplementary Figs. 7–10).

### Detection of structural variation

In order to maximize our discovery of SVs segregating in *F. graminearum*, we included three publicly available chromosome-level assemblies in addition to the PH-1 reference genome in our analyses. Isolates FG-12, CML3066, and CS3005 have sequence matching the 15ADON genotype and were collected from China, Brazil, and Australia, respectively. Pairwise genome comparisons of the seven query assemblies with the PH-1 reference using the software SyRI identified both SNPs and SVs. Isolate FG-12 is unusual in terms of its high divergence from the other isolates in this study based on a neighbor joining tree constructed using SNPs (Supplementary Fig. 1). FG-12 is also very different from the rest of the isolates in terms of SVs. It was isolated in China from maize seedling roots with root rot symptoms. Isolate 23473 does not have nearly as many unique SNPs as FG-12, but it nevertheless groups with it in the tree and has one of the highest totals of SVs (in particular, inversions and translocations). In contrast, isolate 23468 is most similar to the reference isolate PH-1 in terms of SNPs and tends to have fewer SV differences from PH-1 relative to the other isolates.

**Fig. 2.**
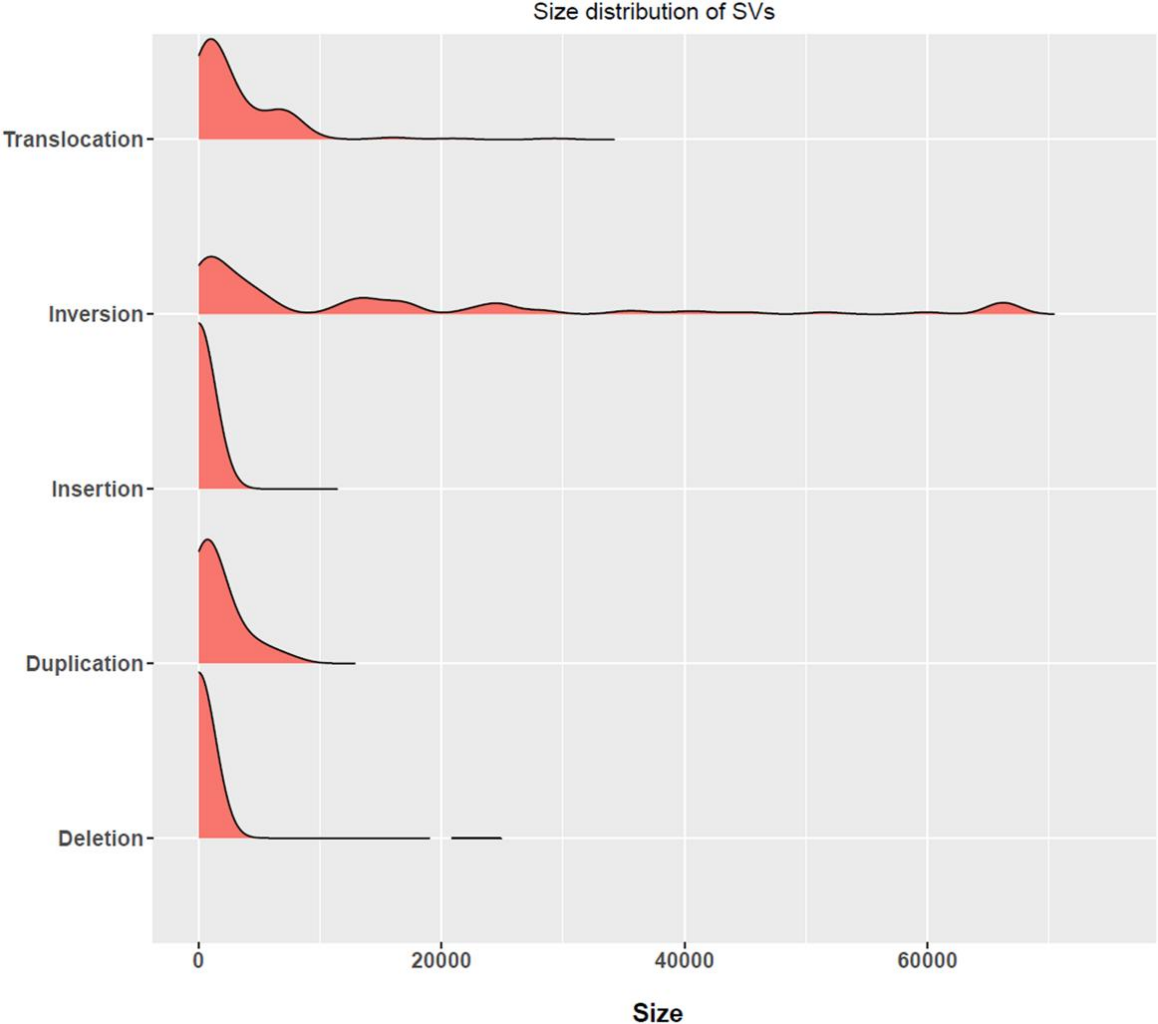
Size distribution of SVs in *F. graminearum* genomes. The following are not included in the plot: artifactual duplications caused by the incomplete assembly of ribosomal RNA repeats at the C-terminus of chromosomal 4, and because of their size (>100 kb), the largest inversion at the end of chromosome 1 in FG-12 and the two largest translocations on chromosome 4 in isolate CS3005.

Excluding insertions and deletions smaller than 1 kb in size and all deletions from FG-12 (see below), we identified a total of 568 SVs (Table 2 and Fig. 1). Inversions were fewer in number but were generally larger in size (Fig. 2). On average across genomes, rearrangements (inversions, translocations, and duplications) were enriched 3.3-fold within 1 Mb of the chromosomal ends (*P* < 0.001 in each genome). When including SVs of all sizes, 32% of inversions, 58% of translocations, 40% of duplications, 38% of insertions, and 35% of deletions originated within 1 Mb of the chromosomal ends in reference isolate PH-1.

**Table 2.** Summary of SVs in *F. graminearum* genomes.

Isolate	Inversions	Translocations	Insertions			Deletions			Duplications
			Total	>1 kb	1 bp	Total	>1 kb	1 bp	
23389	10	28	5,501	14	3,244	7,413	15	4,715	55
23468	4	18	4,102	12	2,177	5,010	9	2,781	50
23473	14	39	6,822	20	3,503	6,788	13	3,370	42
23522	13	20	5,717	15	3,022	5,470	19	2,667	36
CML3066	12	19	5,610	23	2,812	5,168	17	4,292	30
CS3005	5	15	4,605	14	2,181	4,253	10	1,939	21
FG-12	29	20	26,132	18	16,930	NA	NA	NA	11
Total	87	159	58,489	116	33,869	34,102	83	19,764	245

*Note*. Duplications on the end of chromosome 4 are not included as the duplications are artifacts and were identified due to incomplete assembly of repeat regions in query genomes. Deletions in FG-12 are not included.

Summing across each comparison of the seven query genomes with PH-1, we identified a total of 87 inverted regions ranging from 156 bp to over 2.6 Mb (Table 2). Thirty-six inversions are unique to a single isolate, while another 16 are shared among multiple isolates, leading to 52 inversion-state differences when compared to PH-1. Twenty-five distinct inversions are over 10 kb in size, including half of the shared inversions. FG-12 has the most unique and total inversions, while 23473 has the second-most inversions. Eight inversions are common between 23522 and CML3066 and only one inversion each is shared between 23468 and isolates FG-12 and CS3005. The distribution of inversions per isolate ranged from four in 23468 to 29 in FG-12 (Table 2). Of all the inversions, 2, 4, 5, 2, 1, and 1 are unique in 23522, 23473, 23389, CML3066, 23468, and CS3005, respectively. Isolate FG-12 has 21 unique inversions, of which nine are larger than 10 kb, including the largest inversion identified in this study (2,620,177 bp on chromosome 1, corresponding to 2,652,877 bp in the PH-1 reference). Inversion rearrangements are sparse in chromosomes 1 and 3 (a total of 20) but common in chromosomes 2 and 4 (67 in total). Inversions made up 0.09% of the structural rearrangements but covered 9.4% of the size of the PH-1 genome.

There are a total of 159 translocations in the seven query genomes (Table 2). The translocations ranged from 104 bp to 376,520 bp in size, 100 of them being larger than 1 kb. Eighty-five of the translocations are inverted translocations. Sixty-nine translocations are within the same chromosome while 90 are between different chromosomes. Isolate 23473 has the most translocations, while CS3005 has the two largest translocations, 376,520 bp and 152,145 bp in size. The two regions are in chromosome 3 in CS3005 but in chromosomes 1 and 2, respectively, in PH-1. As with inversions, isolates share some translocations in common. However, while translocations identified in different isolates often overlap, the specific positions defining the translocation intervals rarely match, making the identification of translocations inherited from a common ancestor difficult. About a quarter (42) of the translocations originate within 20 kb and about half (93) within 1 Mb from the chromosomal ends in PH-1. Translocations comprised 0.17% of the structural rearrangements. Among the query isolate—PH-1 comparisons, translocations usually cover well under 1% of the size of the PH-1 genome, except for the comparison with CS3005, where translocations cover 1.65% of the genome size (Supplementary Table 3).

SyRI identified a total of 58,489 insertions and 34,102 deletions in the seven query genomes (Table 2). All deletions from FG-12 are excluded from the above count and other analyses as SyRI identified many deletions in FG-12 corresponding exactly to intronic sequences missing in this genome assembly, likely due to some technical error that appears to only affect this one type of SV. Single base pair events made up 58% of the insertions and 53% of the total deletions. A total of 26,132 insertions and 16,930 one bp insertions are from a single isolate, FG-12. Insertions and deletions larger than 1 kb are much less common, with each isolate having 23 or fewer of each type (Fig. 2 and Table 2). The largest deletion, 22,908 base pairs in size in chromosome 2, is shared by isolates 23389 and 23473. This deletion removes the polyketide synthase (PKS6) gene from the fusaristatin A biosynthesis gene cluster and part of the non-ribosomal polyketide synthase (NRPS7) gene from the same cluster. Similarly, another deletion of 4,658 bp in isolate 23473 affects the PKS2 biosynthesis cluster on chromosome 4. Two genes from this cluster (FGRAMPH1_01G16115 and FGRAMPH1_01G16117) are missing and another gene (FGRAMPH1_01G16119) from the same cluster is partially missing. Likewise, the largest insertion identified is 24,017 bp in size in chromosome 1 of isolate FG-12. Even though insertions and deletions made up 62% and 36% of the total count of structural rearrangements, they only covered up to 0.53% and 0.34% of the size of the PH-1 genome in any given PH-1-query isolate comparison (Supplementary Table 3).

The region of ribosomal RNA repeats in the subtelomeric region of chromosome 4 was not completely assembled into contigs in any of the query isolates. Thus, in the SyRI comparisons of PH-1 to each query isolate, the partially assembled repeat regions are misidentified as duplications in PH-1 (Fig. 1, regions at the end of chromosome 4). Aside from these artifactual duplications, SyRI identified from 11 to 55 duplications in each query genome (Table 2 and Supplementary Table 6).

As SVs already appeared enriched in subtelomeric regions, we checked the overlap of structural rearrangements with regions of high recombination (Laurent *et al*. 2018) that include regions at or near each chromosome end. There are a total of 12 such regions, four in chromosome 1, three each in chromosomes 2 and 4, and two in chromosome 3, and these regions cover 29.6% of the genome. Nearly all types of SVs are enriched in these regions of high recombination, as a total of 40.2% of inversions, 44.7% of translocations, 53.5% of insertions (>50 bp), 50.6% of deletions (>50 bp in size, excluding FG-12), and 23.7% of the duplications fall within the regions (Fig. 1). Averaging across six isolates, the rearrangements (inversions, duplications, and translocations) were enriched 2.6-fold in the regions of high recombination (*P* < 0.05 for five of the six genomes). The proposed ancient chromosomal fusion sites overlap and extend beyond the regions of high recombination rate in this fungus (Ma *et al*. 2010; King *et al*. 2015). It is likely that the regions of high recombination extend beyond the current boundaries, as markers used for the construction of the genetic map used to define these regions did not cover the chromosomal ends. If this were true, more SVs would fall in the high recombination regions of the genomes.

**Fig. 3.**
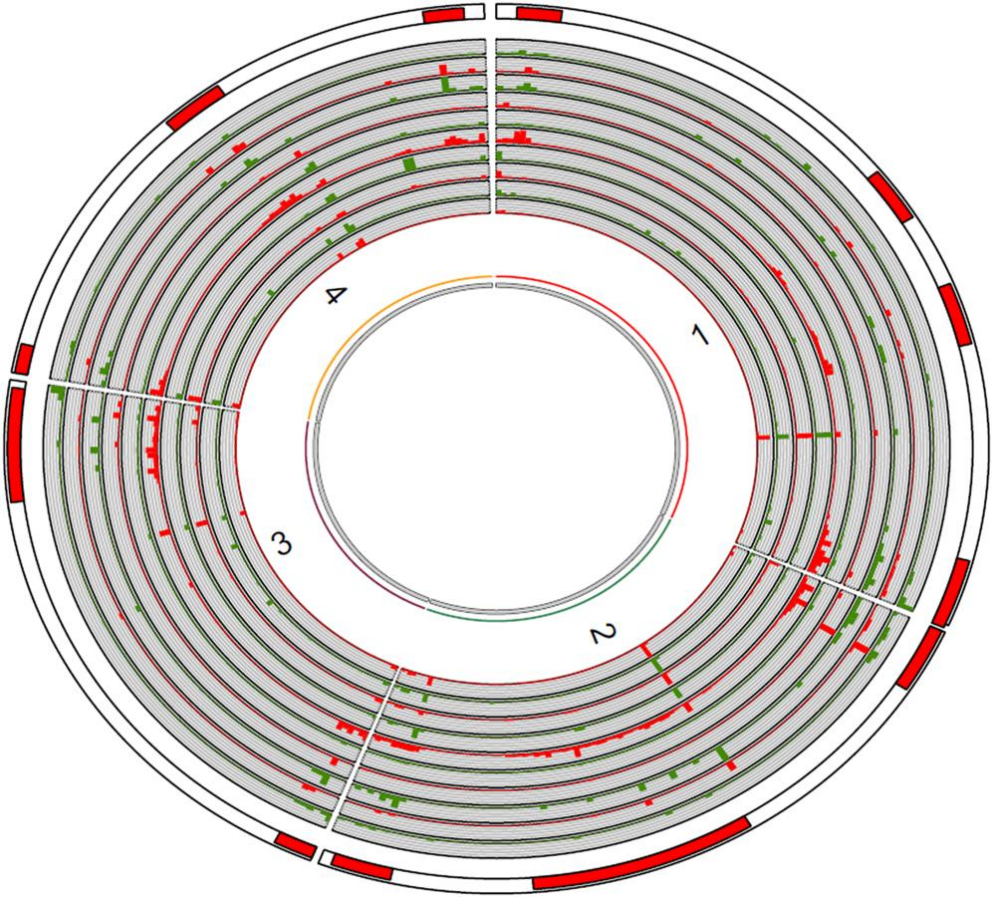
Overlap of different features in *F. graminearum* isolate 23389. Tracks 1–10 consist of the total number (including all sizes) of all events or sum of the lengths of events falling within each non-overlapping 50 kb window. Track 1 (from inside): TE/repeat element count. Track 2: total TE/repeat element length. Track 3: structural rearrangement (inversion, translocation, and duplication) count. Track 4: structural rearrangement length. Track 5: single nucleotide polymorphism (difference from PH-1) count. Track 6: insertion and deletion (indel) count. Track 7: indel length. Track 8: highly diverged region (HDR) count. Track 9: HDR length. Track 10: Orphan contig alignment length. Track 11: regions of high recombination.

*F. graminearum* isolates differ in terms of the types and total number of TEs present in the genome (Table 3 and Supplementary Table 13). The repeat content in the eight *F. graminearum* genomes included in this study ranged from 0.98% in PH-1 to 1.98% in 23473 (Table 3). The EDTA pipeline did not identify gypsy retrotransposons in 23473, PH-1 and CS3005. Tc1_mariner transposons are present in all genomes except FG-12, and the Hat type of DNA transposon is only present in 23522, 23473, 23468, and CS3005. When present, gypsy retrotransposons are present in multiple copies, with 23522 and CML3066 having 114 and 56 copies, respectively. Isolates 23389 and FG-12 have three copies and 23468 has four copies. A few of the gypsy retrotransposons are intact in the genomes: three in 23389, four each in 23468 and 23522, and two in CML3066 (Supplementary Table 14). Three classes of DNA transposon: CACTA, mutator, and Helitron, are present in multiple copies in all genomes. All of the mutator DNA transposons in all genomes were identified as structurally intact. Most of the Tc1-mariner (except one in PH-1) and multiple copies of DNA transposons are intact. A total of 7.3% of insertions, 7.1% of deletions (excluding deletions in FG-12) over 50 bp in size, 38.6% of inversions, 63.9% of translocations, and 57.7% of the duplications overlap with the repeat content of the genome by at least a single base pair (Fig. 3 and Supplementary Figs. 2–6). When the overlap of structural rearrangements with TEs is required to cover over half of the length of both TE and rearrangement, then across isolates approximately 30% of translocations and duplications correspond to an annotated TE, while a smaller proportion of insertions at least 50 bp long (<5%) correspond to TEs. These results indicate that repeat elements are shaping the landscape of SVs in *F. graminearum* genomes. Fisher's exact test identified that rearrangements (inversions, translocations, and duplications) were enriched on average 3.5-fold in the repeat rich regions of the genomes (*P* < 0.05 for all six genomes). Some of this enrichment is due to the one-to-one correspondence between TEs and some identified rearrangements, but additional enrichment of rearrangements in these repeat regions is evident. Likewise, the repeat elements were enriched 2.4-fold in the regions of high recombination (*P* < 10^−4^ in each genome) and 2.1-fold on the subtelomeric regions (1 Mb from chromosomal ends, *P* < 0.01 for all six genomes).

**Table 3.** Summary of TEs/repeat content in *F. graminearum* genomes used in this study.

Isolate	Description	LTR		TIR				Non-TIR		Total
		Gypsy	Unknown	CACTA	Mutator	Tc1_ mariner	Hat	Helitron	Repeat regions	
PH-1	No. of repeat elements	NA	45	46	20	1	NA	10	260	382
	% of genome masked	NA	0.19	0.17	0.12	∼0	NA	0.19	0.31	0.98
23389	No. of repeat elements	3	131	55	23	2	NA	13	361	588
	% of genome masked	0.06	0.48	0.22	0.14	0.01	NA	0.3	0.42	1.63
23468	No. of repeat elements	4	122	51	26	2	32	17	288	542
	% of genome masked	0.08	0.45	0.21	0.16	0.02	0.11	0.31	0.53	1.87
23473	No. of repeat elements	NA	NA	46	26	1	1	16	448	538
	% of genome masked	NA	NA	0.2	0.17	0.01	0.01	0.37	1.22	1.98
23522	No. of repeat elements	114	154	34	30	2	1	14	193	542
	% of genome masked	0.28	0.45	0.2	0.2	0.01	0.01	0.33	0.46	1.94
CML3066	No. of repeat elements	56	45	60	22	1	NA	10	362	556
	% of genome masked	0.08	0.22	0.16	0.14	∼0	NA	0.2	0.74	1.54
CS3005	No. of repeat elements	NA	172	53	19	1	1	8	442	696
	% of genome masked	NA	0.33	0.15	0.12	∼0	0.01	0.14	0.41	1.16
FG-12	No. of repeat elements	3	340	23	21	NA	NA	9	284	680
	% of genome masked	∼0	0.97	0.17	0.15	NA	NA	0.27	0.15	1.71

## Discussion

In this study, we sequenced four representative isolates from *F. graminearum* populations using Oxford Nanopore sequencing and obtained chromosomal-level assemblies. These four genomes and four publicly available genomes, including the established PH-1 reference, were used for the analysis of SVs with the objective of identifying their distribution and role in shaping host–pathogen interactions and genome evolution. We detected a total of 87 inversions, 159 translocations, 245 duplications, 58,489 insertions, and 34,102 deletions. The general features, distribution across the genomes, predictors, and potential roles of SVs in adaptation and evolution are discussed.

Low GC content such as in the regions of short sequence repeats and inactivated transposons, low gene content, recombination rate, and the presence of TEs are the major predictors of structural rearrangements in *Arabidopsis* and *Z. tritici* (Badet *et al*. 2021). Double-stranded breaks during meiosis leading to the intra-chromosomal or inter-chromosomal rearrangement of segments and the movement of DNA segments by transposons are the major sources of large-scale chromosomal variation (Desjardins 2006; Stukenbrock and Croll 2014; Mehrabi *et al*. 2017; Priest *et al*. 2020). Other sources include unequal crossover in repeat regions and double-stranded breaks during the mitotic cycle. All of these factors likely serve as predictors of structural rearrangements in *F. graminearum* as well. At a very broad scale along chromosomes, recombination also seems to be a primary driver of structural rearrangements in *F. graminearum* genomes. The *F. graminearum* genome has regions of widely different average recombination rates, a “two-speed” recombination landscape that is associated with differences in nucleotide diversity and density of host–pathogen interaction genes (Laurent *et al*. 2018). High recombination regions are characterized by high nucleotide diversity and enriched with genes predicted to code for secreted effectors or have a role in secondary metabolite synthesis. Along chromosomes, meiotic crossovers show a positive correlation with gene density and negative correlation with GC content (Laurent *et al*. 2018). Genes reported to show host-specific expression are enriched by 2-fold in the high recombination regions, where high recombination likely shuffles the genes with roles in plant–microbe interaction and secondary metabolite synthesis, promoting rapid evolution (Ma *et al*. 2010). Our results show that recombination is not only associated with high nucleotide diversity and HDRs but also associated with large-scale SVs (Fig. 3 and Supplementary Figs. 2–6). Structural rearrangements are enriched 2.6-fold in the regions of high recombination. A total of 40.2% of inversions, 44.7% of translocations, 53.5% of large insertions (>50 bp in size), 50.6% of large deletions, and 23.7% of the duplications fall within the regions of high recombination (Talas *et al*. 2016), which are also sites of ancient chromosomal fusions in the ancestor to *F. graminearum*. The orphan contigs also tend to map to these regions of high recombination (Fig. 3 and Supplementary Figs. 2–6). This makes recombination critically important for adaptation in this fungus. *F. graminearum* is a homothallic fungus, but field isolates undergo frequent outcrossing in nature (Talas *et al*. 2016), and on the hypothesis that recombination is mutagenic, recombination occurring during both selfing and outcrossing can help drive the evolution of this fungus.

TEs mediate mutations and chromosomal rearrangements, and are key in shaping genome architecture and gene content in many fungal genomes (Stukenbrock and Croll 2014). The repeat content in *F. graminearum* genomes is very low (0.98–1.98%) compared to 24% in *Fusarium oxysporum* (Ma *et al*. 2010), 44.8% reported in different *Colletotrichum* species (Rao *et al*. 2018), and 11% in *M. oryzae* (Chiapello *et al*. 2015). This low repeat content is likely related to repeat induced point mutation (RIP), a process in *F. graminearum* and some other fungi that suppresses duplication and TE-mediated genome expansion (Cuomo *et al*. 2007). Both intact and partial remains of TEs were identified in this study. While the majority of the mutator, Tc1-mariner, CACTA, and helitron TEs identified are intact, only 7% of the Gypsy transposons are intact, suggesting RIP is rapidly mutating gypsy transposons in the genome (Supplementary Table 14). A significant proportion of inversions (38.6%), translocations (63.9%), and duplications (57.7%) overlap with the repeat content of the genome. At a 50 kb window scale, structural rearrangements are 3.5-fold enriched in repeat rich regions, indicating the role for TEs in structural rearrangements. The finding of both high SNP density and an enrichment of SVs in regions of high recombination and high repeat density suggests that recombination and TEs are associated with the origin of small and large-scale genomic variants in *F. graminearum*.

SVs in *F. graminearum* genomes have numerous consequences for gene content, their chromosomal locations, and their coding sequences. A large proportion of the rearranged segments include genic regions annotated in the PH-1 genome. A total of 40.5% of insertions (>50 bp), 47.6% of deletions (>50 bp), 71.3% of inversions, 15.9% of duplications, and 21.4% of the translocations either include at least one gene inside the rearranged segment or have at least one of the breakpoints overlapping a gene (Supplementary Tables 8–11). In particular, translocations have helped in shuffling the location of genes coding for effector proteins and have thus helped in the evolution of interactions in this fungus with its host. Effectors are small, often secreted molecules that modulate plant–microbe interactions. Recognition of effectors by plant resistance genes triggers an immune response, so plant pathogens deploy several strategies to avoid recognition. One of the mechanisms is to lose or mutate recognized effector proteins. We identified some effector-rich regions being moved around the genome through translocation events. Two chromosomal regions in isolate CS3005 and one region in FG-12 containing genes coding for predicted secreted effectors are shuffled relative to the reference PH-1 genome by translocation events. A 372 kb region present in chromosome 1 in PH-1 and in chromosome 3 in CS3005 contains five predicted effector genes (Supplementary Tables 4 and 10). One of the predicted effector genes in this region codes for KP4 killer toxin, which is expressed during wheat seedling infection, and similar proteins are necessary for virulence during head infection (Lu and Faris 2019). Similarly, another 152 kb region in chromosome 3 in CS3005 is present in chromosome 2 in PH-1 and contains five predicted effector genes (Supplementary Tables 4 and 10). A smaller 4.5 kb region in isolate FG-12 is located in a different region within chromosome 1 relative to the reference genome (Supplementary Table 5). Shuffling of effector-rich regions could change the expression profile of the effector proteins and facilitate effector gain and loss. Recombination of strains having identical effectors in two separate genomic locations can result in progeny having multiple or no copies of the gene.

SVs can also directly create presence–absence or copy number variation in fungal effector proteins and thus have a role in shaping host specialization and plant–microbe interactions in many patho-systems (Wang *et al*. 2020; Hou *et al*. 2023). We identified that some predicted effector genes have been lost, which could have been facilitated by translocations as discussed earlier. Deleted segments (>50 bp) in six query isolates partially or completely overlap a total of 229 genes in the PH-1 genome (Supplementary Table 12). Of these genes, 27 are entirely missing in query isolates (Supplementary Table 12). Some missing genes are common between isolates. For example, the gene FGRAMPH1_01G08535, which codes for a predicted secreted effector, a hydrolase enzyme, is missing in isolates 23389 and 23473 (Supplementary Table 12; Brown *et al*. 2012). Abundant insertions relative to PH-1 were detected in our analyses, and BLAST searches of these inserted sequences against the orphan protein database from Kelly and Ward (2018) detected predicted effector proteins. For example, a shared insertion common to the isolates 23389, 23473, 23522, and FG-12 likely consists of a cytochrome P450, a predicted secreted protein and a kinase-like protein. Similarly, a 3 kb insertion in isolate 23468 (23468_666) likely is a cluster of secreted proteins as it consists of predicted secreted proteins (identified by SignalP), pectate lyase, and a lysin domain protein. Insertions consisting of only lysin motif (lysM) containing proteins were also identified in isolates CML3066 (CML3066_888) and CS3005 (CS3005_889). LysMs are about 50 amino acids long and bind to peptidoglycan, chitin, and their derivatives and can help the fungus to evade plant defense responses by binding to the chitin fragments released during enzymatic digestion of plant cell wall during the entry of the fungus into the host cells and/or protecting the fungal cell wall against hydrolytic enzymes (Dubey *et al*. 2020). Similarly, pectin lyase is an enzyme for degrading pectin in the plant cell wall. In addition, the BLAST results of an insertion in isolate 23473 (23473_831) includes a hit that covers 45% of an orphan protein, and the amino acid sequence of that orphan protein is a 29% match to the stocks4 gene. The stocks4 gene was predicted to produce a secreted protein with high confidence by SignalP. Besides complete loss of genes in some isolates, smaller deletions fall within coding sequences of effectors predicted by Brown *et al*. (2012) and Rampitsch *et al*. (2013) and can have important functional consequences as they can make a nonfunctional protein product or help the pathogen evade recognition by its host (Hou *et al*. 2023). Genes FGRAMPH1_01G00015, FGRAMPH1_01G03877, FGRAMPH1_01G08677 in chromosome 1, FGRAMPH1_01G11565, FGRAMPH1_01G12735, FGRAMPH1_01G14149 in chromosome 2, and FGRAMPH1_01G16399 in chromosome 3 have parts of coding sequences missing compared to PH-1 (Supplementary Table 12). Our finding of abundant presence–absence variation further supports the view that structural variation is an essential component of plant–microbe evolution.

Secondary metabolite synthesis clusters are highly diverse in fungi, and this diversity is due to the loss of the clusters/genes mediated by complex structural rearrangements or through gain of clusters via horizontal gene transfer (Massonnet *et al*. 2018; Tralamazza *et al*. 2019). Our analysis predicts that the fusaristatin A and PKS2 biosynthesis gene clusters are nonfunctional in some isolates. The fusaristatin A gene cluster is partially deleted in *F. graminearum* isolates 23389 and 23473 and falls within the largest deletion observed in this study, a 22,098 bp deletion in chromosome 2 (Supplementary Tables 7 and 11). Among the two genes in the cluster, the PKS6 is absent, and the non-ribosomal peptide synthase (NRPS7) is truncated in these two isolates. Natural populations of *Fusarium pseudograminearum* having similar deletion (PKS6 missing and NRPS7 truncated) in this biosynthetic gene cluster exist in western Australia and are more aggressive pathogens causing crown rot of wheat (Wollenberg *et al*. 2019; Khudhair *et al*. 2020). The experimental deletion of PKS6 and the disruption of NRPS7 in two isolates of *F. pseudograminearum* increased the growth rate and aggressiveness of the mutants toward wheat (Khudhair *et al*. 2020). The role of PKS6 in the *F. graminearum* reference isolate PH-1 has been previously investigated by the generation of a gene disruption mutant. Disruption of PKS6 in the PH-1 background significantly increased growth rate but did not significantly change pathogenicity toward a susceptible wheat cultivar (Gaffoor *et al*. 2005). The difference in pathogenicity test results among the two studies could be due to the difference in methods and genetic backgrounds of the isolates. Gaffoor *et al*. (2005) only disrupted the PKS6 gene of the cluster while Khudhair *et al*. (2020) completely deleted PKS6 and disrupted NRPS7. It is also possible that the cluster is important for crown rot disease and not FHB. The fusaristatin A biosynthesis genes in *F. graminearum* (PH-1) and *F. pseudograminearum* (CS5834) share 96.2% nucleotide identity, and the role of the loss of this gene cluster in the genetic background of more *F. graminearum* isolates should be investigated. Similarly, two of the 18 genes of the PKS2 polyketide synthesis gene cluster were fully deleted and one gene was partially deleted in isolate 23473 (del 4110 in Supplementary Table 5). The deleted genes, FGRAMPH1_01G16115 and FGRAMPH1_01G16117, are RTA1-like protein and RTA1 domain containing protein, and the truncated gene FGRAMPH1_01T16119 is a c6 transcription factor (Zhao *et al*. 2014). Deletions in this gene cluster can have important functional consequences, as strains with a disruption in the PKS2 gene (FGRAMPH1_01G16085) of this gene cluster have a reduced growth phenotype (Gaffoor *et al*. 2005). Our results suggest that structural rearrangements are likely playing a major role in introducing presence–absence variation of secondary metabolite cluster genes in *F. graminearum*.

The identified insertions relative to PH-1 have the potential to code for proteins novel to this species. A total of 46 insertions have translated nucleotide sequences matching one or more sequences in the orphan protein database of Kelly and Ward (2018) with at least 50% coverage and 40% identity. Based on BLAST searches of the orphan proteins, the inserted segments matched, insertions likely include coding sequences for proteins like SNF2 helicase-like protein, arylsulphatase, kinesin light chain protein, and ankyrin repeat containing proteins. SNF2 helicase-like proteins have a role in the mechanical remodeling of chromatin structures by moving the nucleosomes around (Ryan and Owen-Hughes 2011), while arylsulphatases are responsible for sulfur scavenging and influence fungal growth and morphology (Korban *et al*. 2017). Kinesin is a component of the microtubule cytoskeleton. Although kinesin light chains are absent in some fungi (e.g. *Neurospora*; Seiler *et al*. 2000), in humans and higher eukaryotes they are known to play an important role in attaching cargo to kinesin −1 protein (Woźniak and Allan 2006). Kinesin −1 plays a role in moving organelle cargo (endoplasmic reticulum, Golgi apparatus, secretory vesicles, mitochondria, and other cellular organelles) in the cellular space (Wozniak *et al*. 2004). While many accessory genes with presence–absence polymorphisms in *F. graminearum* might not boost fitness in natural environments, some subset may provide a fitness advantage, at least in some environments, and could currently be increasing in frequency toward fixation. These genes that may confer higher fitness could serve important roles in survival, growth, reproduction, fungicide resistance, and virulence.

SVs have not been systematically studied in many plant pathogens. In this study, we identified large SVs in *F. graminearum* genomes, many of which are located toward the chromosome ends and overlap regions of high recombination. TEs and repeat elements are also enriched in the regions of high recombination. We identified presence–absence polymorphisms in biosynthesis gene clusters, predicted effector genes and genes associated with fitness advantages, and plant–microbe interaction. We also identified that genomic regions with roles in plant–microbe interactions (predicted effectors) are shuffled in different genomic regions by structural rearrangements and cases where breakpoints of the rearrangements overlap coding sequences. To summarize, SVs in *F. graminearum* have an important role to play in shaping pathogen–host interactions and broader evolution through genome reorganization, the introduction of presence–absence polymorphisms, and changing gene content, protein products, and gene regulation.

## Supplementary Material

jkae065_Supplementary_Data

## Data Availability

Data for this study were submitted to NCBI under BioProject ID PRJNA989623. This includes the four chromosome-level genome assemblies and their gene annotations, the associated BioSample information, and the raw reads that were submitted to the Sequence Read Archive from MinION and Illumina sequencing. Scripts used in the genome assembly pipeline and in other analyses can be found at https://github.com/chris-toomajian/F_graminearum_SVgenomes. Additional data related to the analyses in this work are available at FigShare (10.6084/m9.figshare.c.7100803.v1). Supplemental material available at G3 online.
