# Supplementary material for: The landscape and predicted roles of structural variants in Fusarium graminearum genomes
Source: G3 (Bethesda). 2024 Mar 28;14(6):jkae065. doi: 10.1093/g3journal/jkae065 (PMC11152077; doi:10.1093/g3journal/jkae065)
Supplement: jkae065_Supplementary_Data [file jkae065_supplementary_data.zip › Supplemental_Material_Legends_G3-2024-404872.docx]

**Figure S1:** Neighbor-joining tree for isolates used in the study. Tree was constructed using single nucleotide polymorphisms (SNPs) identified by SyRI in query isolates against PH-1 genome.

**Figure S2:** **Overlap of different features in *Fusarium graminearum* isolate 23468**. Tracks 1 to 10 consist of the total number (including all sizes) of all events or sum of the lengths of events falling within each non-overlapping 50 kb window. Track 1 (from inside): Transposable element/ repeat element count. Track 2: Total Transposable element/ repeat element length. Track 3: Structural rearrangement (Inversion, translocation, duplication) count. Track 4: Structural rearrangement length. Track 5: Single nucleotide polymorphism (difference from PH-1) count. Track 6: Insertion and deletion (indel) count. Track 7: Indel length. Track 8: Highly diverged region (HDR) count. Track 9: HDR length. Track 10: Orphan contig alignment length. Track 11: Regions of high recombination.

**Figure S3:** **Overlap of different features in *Fusarium graminearum* isolate 23473**. Tracks 1 to 10 consist of the total number (including all sizes) of all events or sum of the lengths of events falling within each non-overlapping 50 kb window. Track 1 (from inside): Transposable element/ repeat element count. Track 2: Total Transposable element/ repeat element length. Track 3: Structural rearrangement (Inversion, translocation, duplication) count. Track 4: Structural rearrangement length. Track 5: Single nucleotide polymorphism (difference from PH-1) count. Track 6: Insertion and deletion (indel) count. Track 7: Indel length. Track 8: Highly diverged region (HDR) count. Track 9: HDR length. Track 10: Orphan contig alignment length. Track 11: Regions of high recombination.

**Figure S4:** **Overlap of different features in *Fusarium graminearum* isolate 23522** Tracks 1 to 10 consist of the total number (including all sizes) of all events or sum of the lengths of events falling within each non-overlapping 50 kb window. Track 1 (from inside): Transposable element/ repeat element count. Track 2: Total Transposable element/ repeat element length. Track 3: Structural rearrangement (Inversion, translocation, duplication) count. Track 4: Structural rearrangement length. Track 5: Single nucleotide polymorphism (difference from PH-1) count. Track 6: Insertion and deletion (indel) count. Track 7: Indel length. Track 8: Highly diverged region (HDR) count. Track 9: HDR length. Track 10: Orphan contig alignment length. Track 11: Regions of high recombination.

**Figure S5:** **Overlap of different features in *Fusarium graminearum* isolate CML3066**. Tracks 1 to 10 consist of the total number (including all sizes) of all events or sum of the lengths of events falling within each non-overlapping 50 kb window. Track 1 (from inside): Transposable element/ repeat element count. Track 2: Total Transposable element/ repeat element length. Track 3: Structural rearrangement (Inversion, translocation, duplication) count. Track 4: Structural rearrangement length. Track 5: Single nucleotide polymorphism (difference from PH-1) count. Track 6: Insertion and deletion (indel) count. Track 7: Indel length. Track 8: Highly diverged region (HDR) count. Track 9: HDR length. Track 10: Orphan contig alignment length. Track 11: Regions of high recombination.

**Figure S6:** **Overlap of different features in *Fusarium graminearum* isolate CS3005**. Tracks 1 to 10 consist of the total number (including all sizes) of all events or sum of the lengths of events falling within each non-overlapping 50 kb window. Track 1 (from inside): Transposable element/ repeat element count. Track 2: Total Transposable element/ repeat element length. Track 3: Structural rearrangement (Inversion, translocation, duplication) count. Track 4: Structural rearrangement length. Track 5: Single nucleotide polymorphism (difference from PH-1) count. Track 6: Insertion and deletion (indel) count. Track 7: Indel length. Track 8: Highly diverged region (HDR) count. Track 9: HDR length. Track 10: Orphan contig alignment length. Track 11: Regions of high recombination.

**Figure S7**: Depth and position along chromosomes of orphan contigs from Kelly and Ward (2018) mapped against the genome of isolate 23389. Vertical red lines mark the end of chromosomes 1, 2, 3 and 4.

**Figure S8**: Depth and position along chromosomes of orphan contigs from Kelly and Ward (2018) mapped against the genome of isolate 23468. Vertical red lines mark the end of chromosomes 1, 2, 3 and 4.

**Figure S9**: Depth and position along chromosomes of orphan contigs from Kelly and Ward (2018) mapped against the genome of isolate 23473. Vertical red lines mark the end of chromosomes 1, 2, 3 and 4.

**Figure S10**: Depth and position along chromosomes of orphan contigs from Kelly and Ward (2018) mapped against the genome of isolate 23522. Vertical red lines mark the end of chromosomes 1, 2, 3 and 4.

**Table S1**: Information on isolates sequenced for this study.

**Table S2**: Sizes of each of the four chromosomes for isolates assembled during this study.

**Table S3**: Size, count and proportion of the PH-1 genome affected by different structural rearrangements for each assembly.

**Table S4**: Information (e.g., position in reference and query, size) on inversions identified.

**Table S5**: Information on translocations identified.

**Table S6**: Information on duplications identified.

**Table S7**: Information on insertions greater to or equal to 50 base pairs identified.

**Table S8**: Information on deletions greater to or equal to 50 base pairs identified.

**Table S9**: List of genes in PH-1 partially or completely overlapping Inversions in query genomes, and phenotypes and GO terms of the genes.

**Table S10**: List of genes in PH-1 partially or completely overlapping duplications in query genomes, and phenotypes and GO terms of the genes.

**Table S11**: List of genes in PH-1 partially or completely overlapping translocations in query genomes, and phenotypes and GO terms of the genes.

**Table S12**: List of genes in PH-1 partially or completely overlapping deletions (equal to or greater than 50 base pairs) in query genomes, and phenotypes and GO terms of the genes.

**Table S13**: Transposable elements/repeat content identified and their class and location in *Fusarium graminearum* genomes.

**Table S14**: Summary of Intact Transposable elements/repeat content in *Fusarium graminearum* genomes used in this study.

**Table S15**: Total length of NUCmer alignments of orphan contigs from Kelly and Ward (2018) mapped to *Fusarium graminearum* genomes assembled for this study.
