# Supplementary figures and images for: The landscape and predicted roles of structural variants in Fusarium graminearum genomes
Source: G3 (Bethesda). 2024 Mar 28;14(6):jkae065. doi: 10.1093/g3journal/jkae065 (PMC11152077; doi:10.1093/g3journal/jkae065)

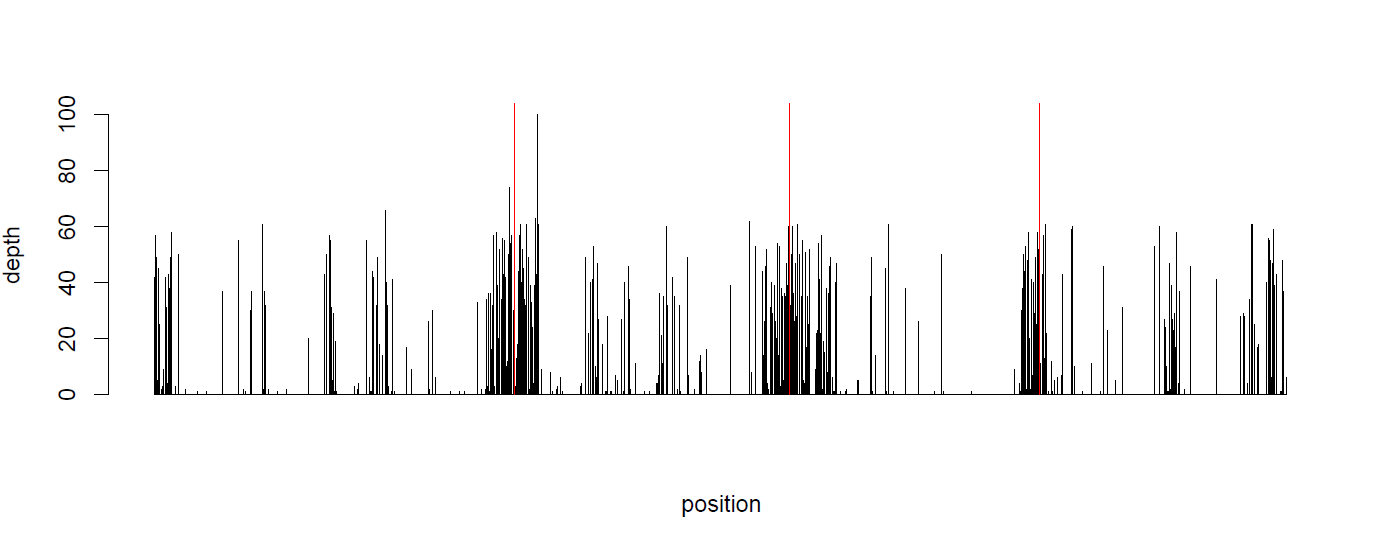

Supplement: jkae065_Supplementary_Data [file jkae065_supplementary_data.zip › Figure_S10_G3-2024-404872.png]

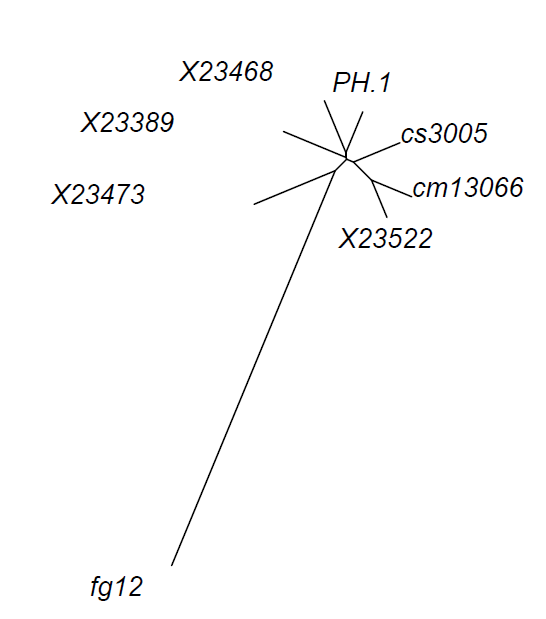

Supplement: jkae065_Supplementary_Data [file jkae065_supplementary_data.zip › Figure_S1_G3-2024-404872.png]

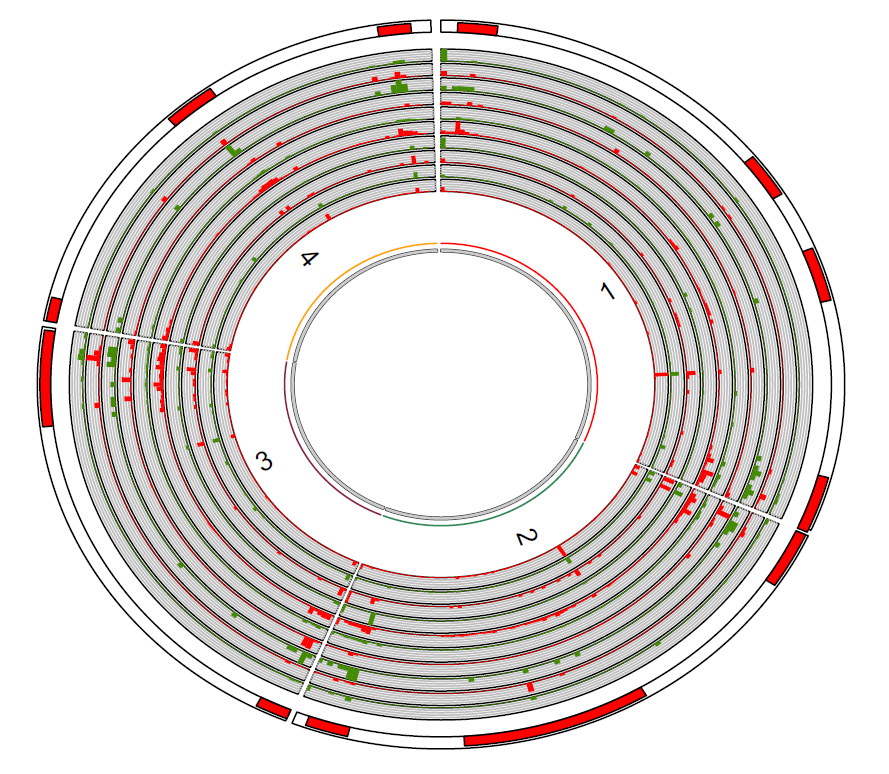

Supplement: jkae065_Supplementary_Data [file jkae065_supplementary_data.zip › Figure_S2_G3-2024-404872.png]

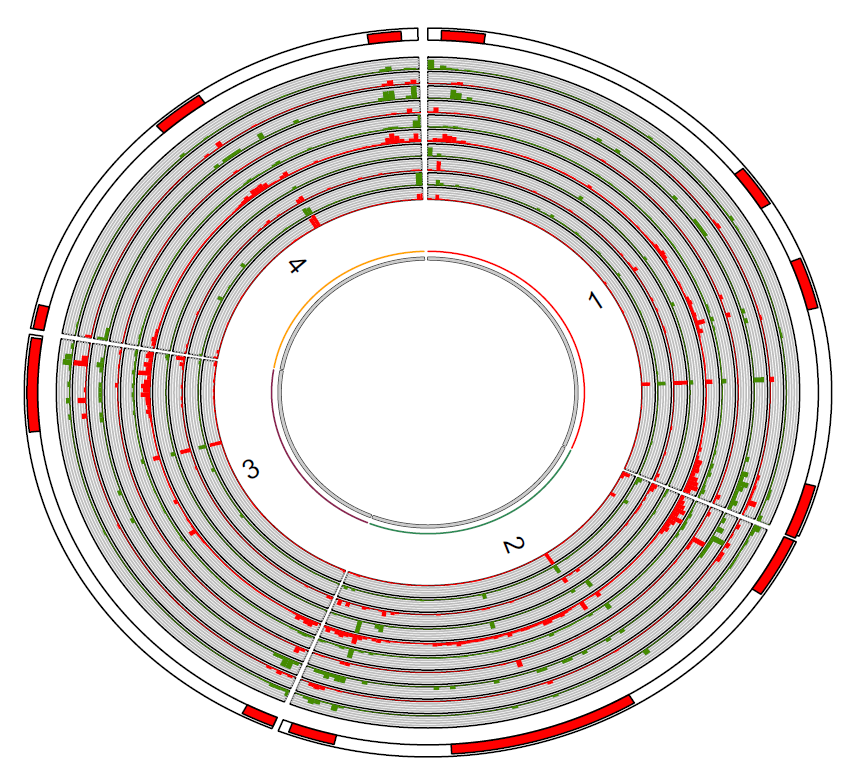

Supplement: jkae065_Supplementary_Data [file jkae065_supplementary_data.zip › Figure_S3_G3-2024-404872.png]

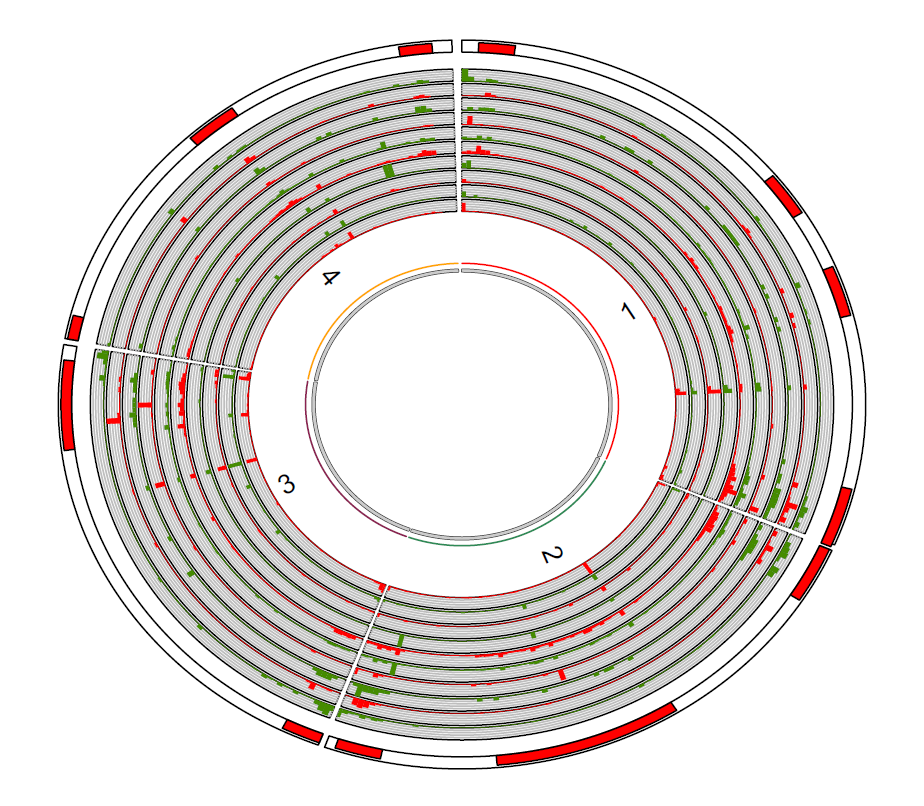

Supplement: jkae065_Supplementary_Data [file jkae065_supplementary_data.zip › Figure_S4_G3-2024-404872.png]

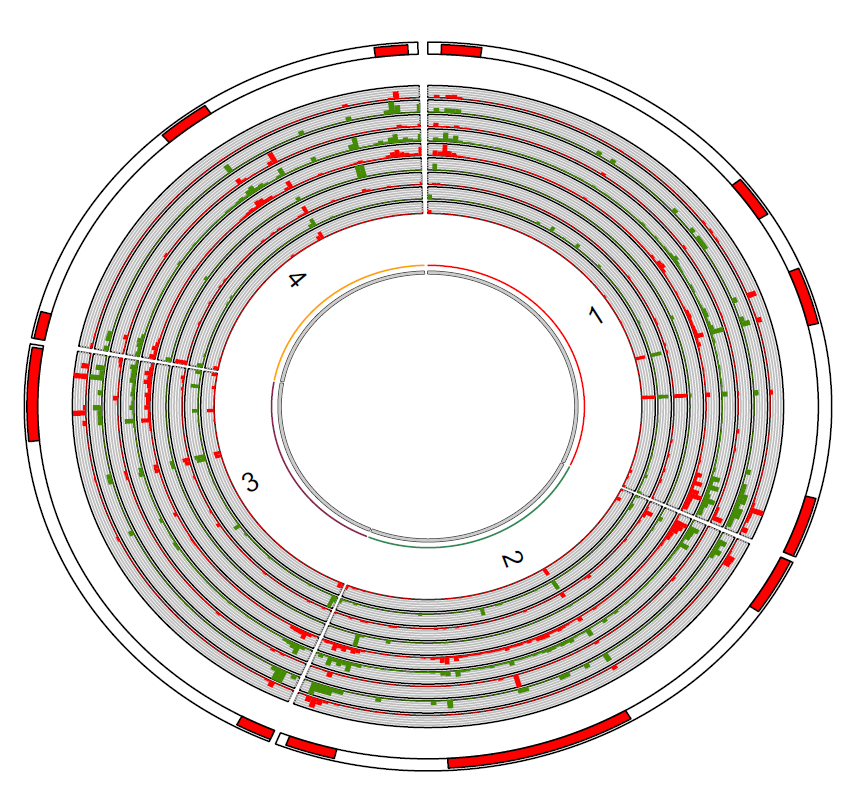

Supplement: jkae065_Supplementary_Data [file jkae065_supplementary_data.zip › Figure_S5_G3-2024-404872.png]

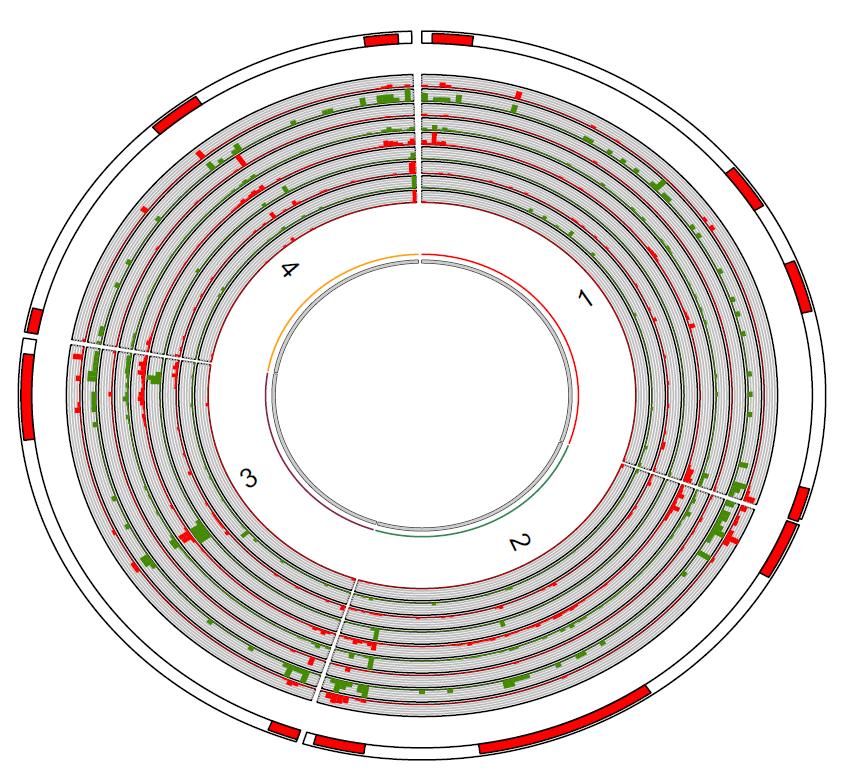

Supplement: jkae065_Supplementary_Data [file jkae065_supplementary_data.zip › Figure_S6_G3-2024-404872.png]

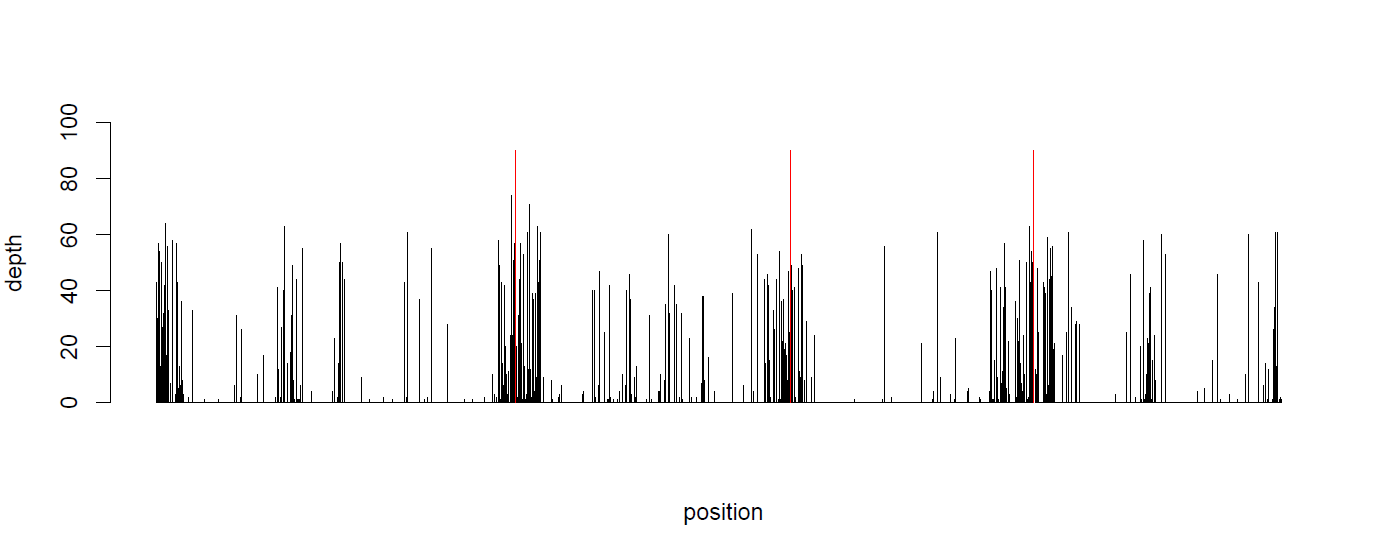

Supplement: jkae065_Supplementary_Data [file jkae065_supplementary_data.zip › Figure_S7_G3-2024-404872.png]

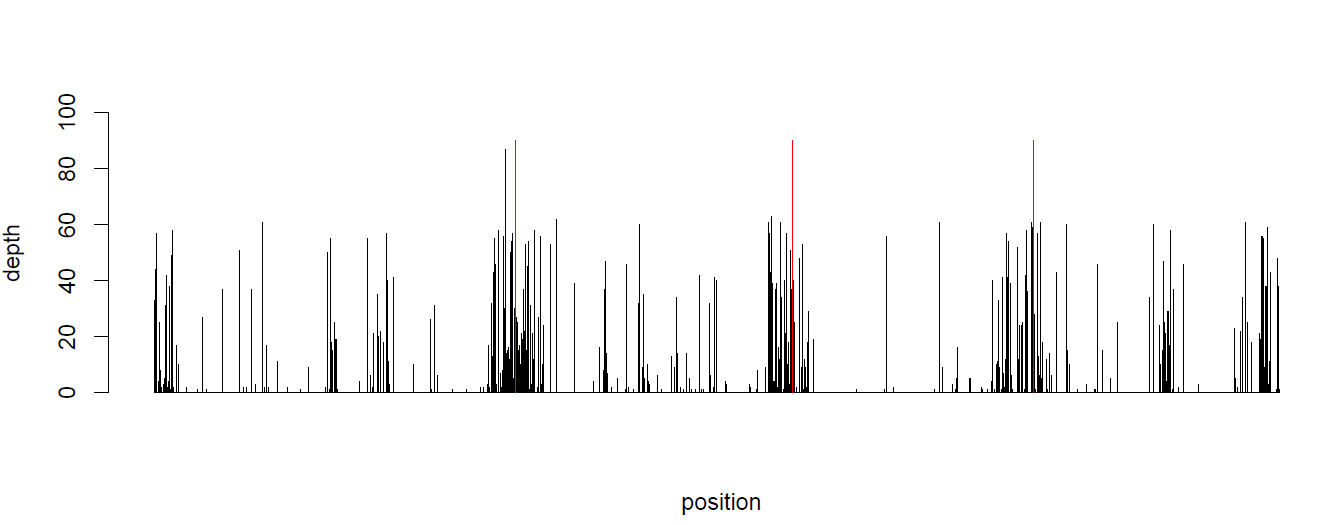

Supplement: jkae065_Supplementary_Data [file jkae065_supplementary_data.zip › Figure_S8_G3-2024-404872.png]

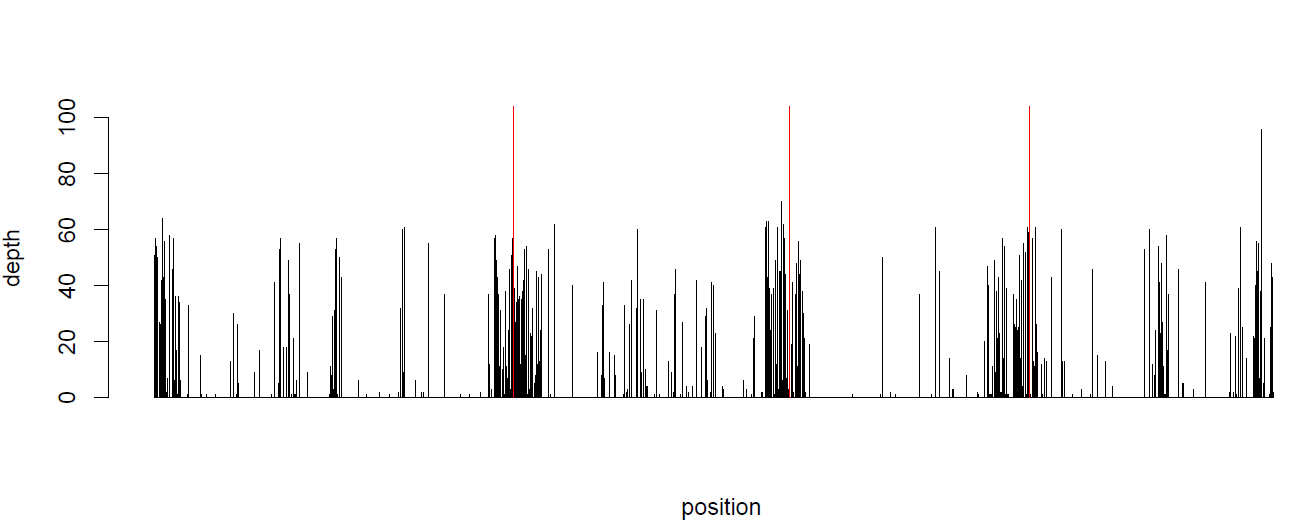

Supplement: jkae065_Supplementary_Data [file jkae065_supplementary_data.zip › Figure_S9_G3-2024-404872.png]
